# Microstructure and Corrosion Resistance of LaNi_5-x_Mg_x_ Alloys

**DOI:** 10.3390/mi13081192

**Published:** 2022-07-28

**Authors:** Krystyna Giza, Edyta Owczarek

**Affiliations:** Faculty of Production Engineering and Materials Technology, Czestochowa University of Technology, Av. Armii Krajowej 19, 42-218 Częstochowa, Poland; krystyna.giza@pcz.pl

**Keywords:** AB_5_ hydrogen storage alloys, magnesium, corrosion resistance

## Abstract

This study analysed the corrosion parameters of LaNi_5-x_Mg_x_ hydrogen-absorbing alloys depending on the degree of replacement of nickel with magnesium and the exposure time of samples in a strongly alkaline solution. The microstructure and composition of the alloys were analysed using SEM and EDS, respectively. A correlation was observed between the corrosion rate and the magnesium content in the alloy and the exposure time of the investigated materials in the corrosive solution. The obtained research results showed that the LaNi_5_ phase, rich in Mg, corroded easily, and the presence of Mg in LaNi_5-x_Mg_x_ alloys became beneficial only for longer exposure times of samples in an alkaline solution. The corrosion layer formed during the contact of the magnesium alloys with the electrolyte promoted faster H_2_ evolution compared to the non-magnesium-substituted alloy.

## 1. Introduction

The prospect of the depletion of traditional energy resources such as oil, coal, and natural gas, as well as the environmental problems (global warming, smog, and dilapidation of human health and plant growth) caused by burning them, have made it necessary to search for sustainable and renewable alternative energy sources. Hydrogen has been recognised as an ideal alternative to fossil fuels, owing to its high energy density (i.e., 120.7 kJ/g) [1], environmentally benign products of oxidation, and abundant content in the universe. Although the techniques for producing hydrogen have become relatively well known in recent decades, highly efficient and safe storage technologies remain a major barrier to this energy source [2,3,4,5,6,7,8].

Intermetallic compounds based on LaNi_5_ (AB_5_-type) alloys are currently widely used as electrodes in nickel–metal hydride (Ni/MH) batteries due to their fast hydrogen absorption, high reversibility, and proper plateau pressures at ambient temperatures [9,10,11,12]. However, the current research on this type of material indicates that LaNi_5_ hydride electrodes are subject to corrosive processes that affect not only lower hydrogen absorption by the alloy, but also the kinetics of hydrogenation and dehydrogenation. Moreover, corrosion also increases water consumption, which causes the separator in the cell to dry out, increasing the cell’s internal impedance [13]. The corrosive processes taking place in a strong alkaline environment on the surface of AB_5_ alloys, along with their irreversible mechanical degradation caused by numerous charging/discharging cycles, are the main cause of the deactivation of hydride electrode materials, and affect the limited efficiency of Ni-MH cells [14,15,16,17]. It was reported that on the surface of the LaNi_5_ electrode, a tight corrosion layer is formed, mainly built of acicular La(OH)_3_ crystals [13], which is a barrier to the processes of hydrogen absorption/desorption.

In order to improve the hydrogen storage properties as well as the corrosion resistance and cyclic stability of LaNi_5_ base alloys, substituting different elemental species into the Ni lattice sites has been widely used [18,19,20,21,22,23,24,25,26,27,28,29,30,31,32]. LaNi_5-x_Mg_x_ alloys seem to be promising due to the advantages of their high capacity deriving from Mg-based hydrogen storage materials [33,34] and excellent kinetics properties resulting from LaNi_5_-based alloys. On the other hand, studies related to the hydrogen-storage properties of LaNi_5_ alloys, in which Ni was replaced with Mg with a content of less than 10 atom %, are rarely described. Recent investigations by T. Li et al. [35] of (LaNi_5_)_1-x_Mg_x_ (x = 0, 0.018, 0.041, 0.063) alloys have shown that the addition of a small amount of Mg alloys can improve the hydriding kinetic property of LaNi_5_ alloys, and (LaNi_5_)_0.982_Mg_0.018_ exhibited the best kinetics. The authors of another work revealed that the hydrogen capacity of the LaNi_4.8_Mg_0.2_ alloy and the low equilibrium pressure of hydrogen make this alloy more attractive as a potential electrode material for Ni-MH batteries than the unsubstituted LaNi_5_ [36]. Nevertheless, there are no references to the influence of magnesium on the corrosive behaviour of La (Ni, Mg) _5_ alloys. It is well known that magnesium is one of the most electrochemically active metals and may deteriorate the corrosion stability of metallic materials in the passive range. Therefore, understanding the corrosion mechanism is extremely important from the point of view of limiting the degradation of the studied hydrogen storage alloys.

The aim of this study was to investigate the effect of the partial replacement of Ni with Mg in hydride electrode LaNi_5-x_Mg_x_ (x = 0, 0.2, and 0.3) alloys on the corrosion properties and evaluate the applicability of the investigated alloys in nickel–hydride cells.

## 2. Experimental Part

### 2.1. Research Material

Studies were carried out for three LaNi_5-x_Mg_x_ alloys (x = 0, 0.2, and 0.3). The alloys were obtained by melting appropriate amounts of metals in an arc furnace under a protective argon atmosphere (La, Ni, Mg, purity 99.99%, Sigma Aldrich, St. Louis, MO, USA). Melting of the constituent elements was carried out in copper crucibles cooled with water at a temperature of about 1500 ℃. After remelting, samples of the alloys were placed in quartz ampoules, a vacuum was created and, after melting, homogenised at a temperature of 600 °C for 300 h. To prevent losses in magnesium during the synthesis, some excess of this metal was used in relation to the assumed stoichiometry of the alloys.

### 2.2. Research Methodology

#### 2.2.1. Optical Microscopy, Scanning Electron Microscopy, and X-ray Microanalysis of EDS Chemical Composition

Samples of metallographic specimens etched in HNO_3_ (65%, 65 mL) + CH_3_COOH (100%, 25 mL) + H_2_O (25 mL) were observed using an Olympus GX51 optical microscope and a JEOL JSM-6610 LV electron microscope. The chemical composition of the studied samples was examined by means of X-ray spectroscopy with energy dispersion (EDS) in randomly selected places on the surface of the samples.

#### 2.2.2. Electrochemical Studies

Polarization measurements were made using a CHI 440A electrochemical measuring device from CH Instruments, Austin, TX, USA in a three-electrode system. The solid alloy samples placed in Plexiglas caps were the working electrodes. The auxiliary electrode was a platinum electrode and the reference electrode was a saturated calomel electrode (SCE). All the measurements were made in a 6M solution of potassium hydroxide at room temperature. The electrolyte solution was in contact with air. Electrochemical tests were performed for all three samples at the following time intervals: 1 h, 24 h, and 66 h. The tests consisted of measuring the open circuit potential (OCP), linear polarization, and plotting polarization curves.

## 3. Research Results

### Analysis of Chemical Composition of Studied Materials

**Figure 1:**
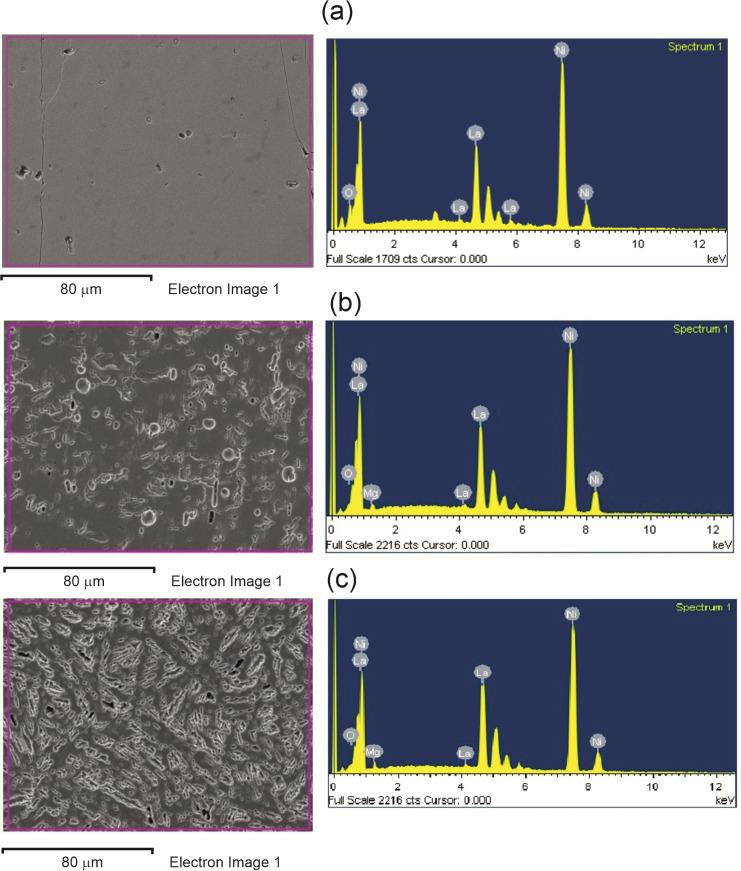
Microstructures and EDS analysis results for alloys: LaNi_5_ (**a**); LaNi_4.8_Mg_0.2_ (**b**); LaNi_4.7_Mg_0.3_ (**c**).

Figure 1 shows the microstructures of the three investigated LaNi_5-x_Mg_x_ alloys. As can be seen, these materials had a dendritic structure in which two areas were visible: dendrites (light etching) and interdendritic spaces (dark areas). In order to determine the elemental composition of the examined alloys (Figure 1), measurements were made using energy-dispersive X-ray spectroscopy (EDS) and scanning electron microscopy (SEM). Table 1 presents the results of chemical composition measurements for the LaNi_5_, LaNi_4.8_Mg_0.2_, and LaNi_4.7_Mg_0.3_ alloys from three different areas. The analysis of the obtained data confirmed the presence of lanthanum, nickel, and magnesium in the investigated metallic materials in amounts corresponding to the LaNi_5-x_Mg_x_ stoichiometry (x = 0, 0.2, and 0.3). The presence of oxygen on the surface of the studied materials proved their high chemical activity.

**Figure 2:**
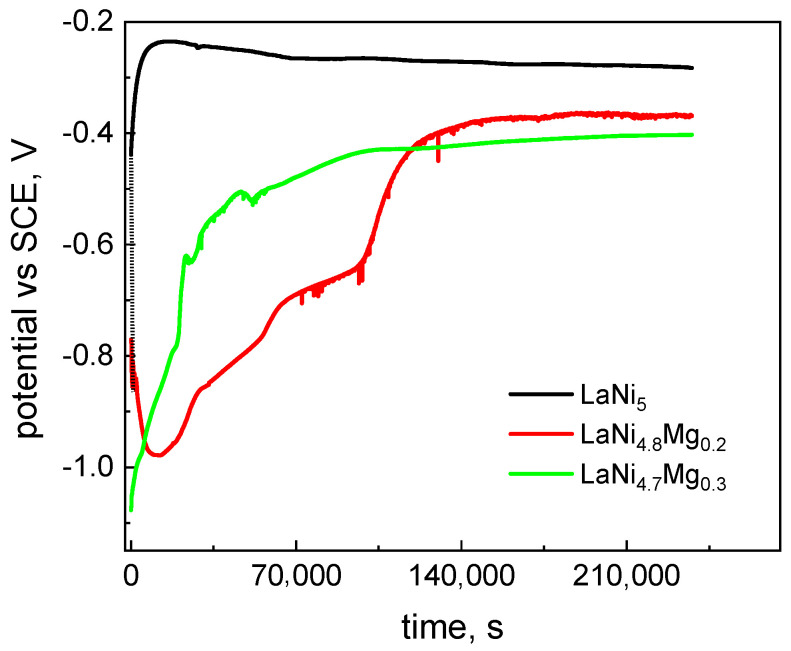
OCP changes for LaNi_5-x_Mg_x_ alloys (x = 0, 0.2, and 0.3) recorded for 66 h.

The corrosion potential of the investigated materials was determined by measuring the open circuit potential (*E*_OCP_) immediately after immersing the electrodes in a 6M KOH solution. Prior to each measurement, the alloy samples were ground with water-based sandpaper, ending with 1200 grit. Figure 2 shows the *E*_OCP_ changes for the examined alloys recorded beginning at the moment the electrodes were immersed in the electrolyte for 66 h. For all the alloy samples, there were noticeable changes in the open circuit potential during exposure to a corrosive solution. For magnesium-substituted alloys, only after a longer time (about 40 h) could we observe a clear stabilization of the potential values. After 66 h of being kept in a corrosive solution, the respective *E*_OCP_ values were: LaNi_5_: −0.28 V, LaNi_4.8_Mg_0.2_: −0.37 V, and LaNi_4.7_Mg_0.3_: −0.40 V.

Based on the conducted *E*_OCP_ measurements, we concluded that the alloys containing magnesium showed a greater tendency to corrosion from the thermodynamic point of view [28,37]. However, thermodynamic susceptibility to corrosion did not provide us with any information on the corrosion rate; therefore, linear polarization measurements were performed in the range of ± 10 mV with respect to the OCP. The measurements were carried out after 1, 24, and 66 h of keeping the materials in a corrosive solution. The Stern–Geary equation was used to determine the value of the corrosion rate (*i*_corr_):

(1) $$R_{p}=\frac{b_{a}b_{k}}{2.3\;(b_{a}+b_{k})\;i_{\mathrm{corr}}}$$

where:

*R_p_* is the polarization resistance;

*b*_a_ and *b*_k_ are the directional coefficients of rectilinear sections of the anode and cathode curves, respectively.

The investigated materials underwent passivation in an alkaline environment; hence, for the anode curve, *b*_a_ → ∞. From this, we finally get:

(2) $$R_{p}=\frac{b_{k}}{2.3\;i_{\mathrm{corr}}}$$

**Figure 3:**
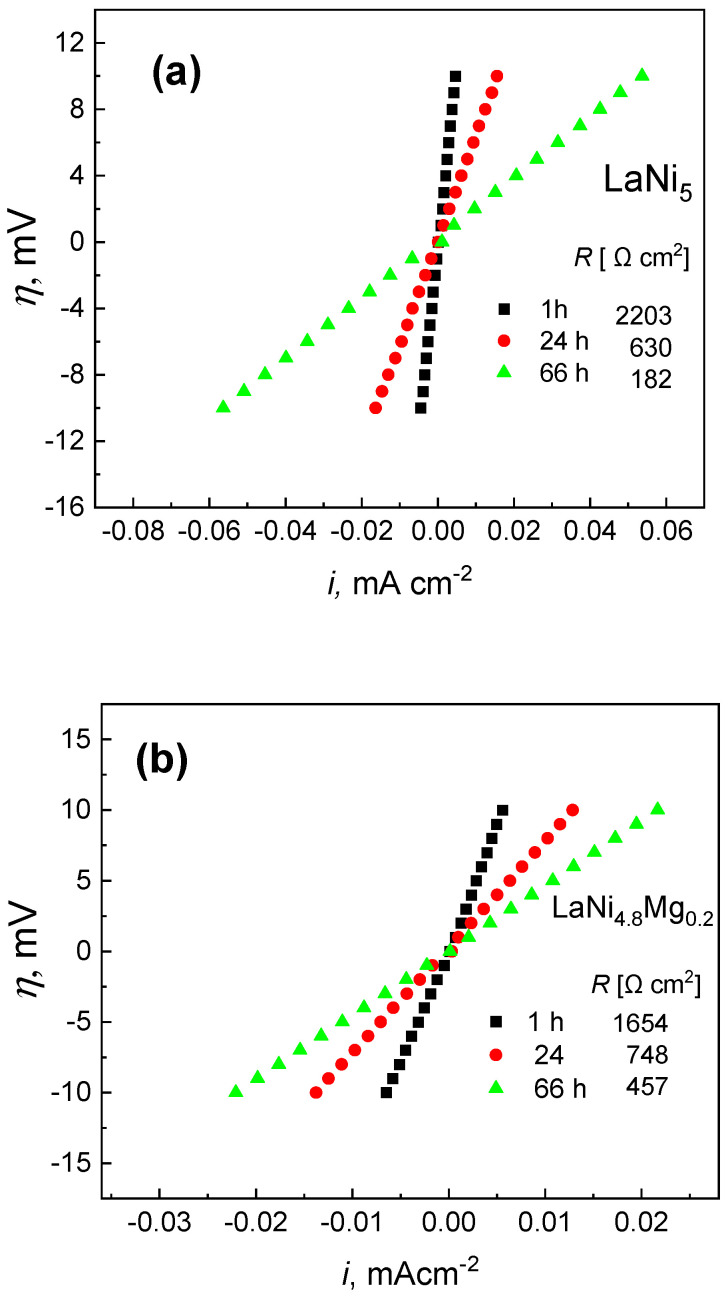

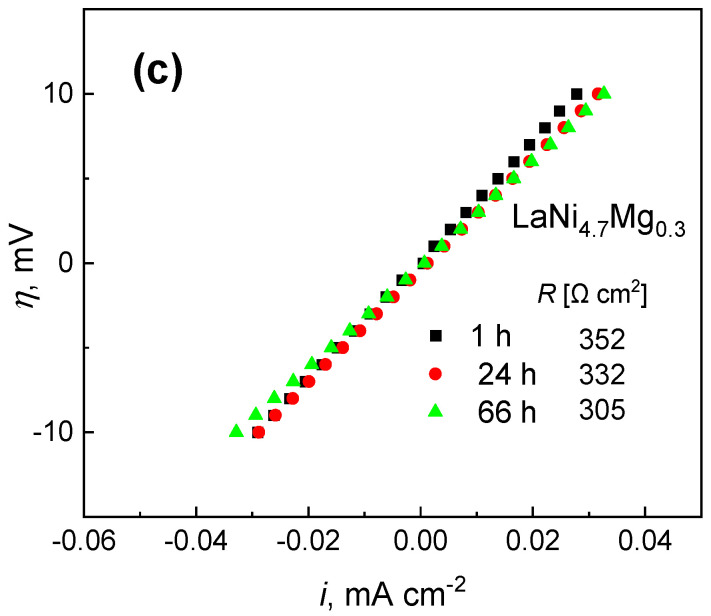
Linear polarization in the range of *E*_OCP_ ± 10 mV for the studied alloys: LaNi_5_ (**a**); LaNi_4.8_Mg_0.2_ (**b**); LaNi_4.7_Mg_0.3_ (**c**).

The slope of the straight lines in Figure 3 is a measure of the polarization resistance (*R*). As shown in Figure 3, the longer the contact time of the electrolyte with the electrode material, the lower the resistance to polarization of the studied alloys (the polarization resistance decreased). Only the LaNi_4.7_Mg_0.3_ alloy did not show a significant change in the polarization resistance.

**Figure 4:**
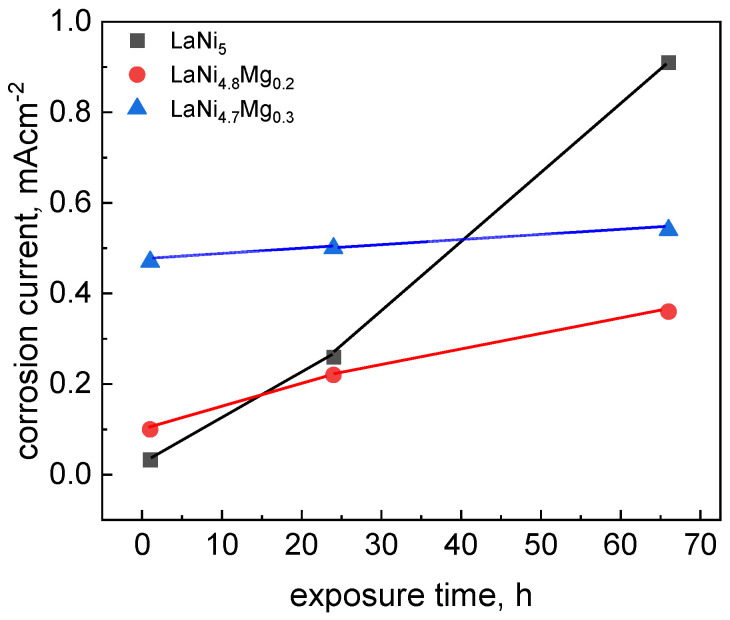
Corrosion rate dependence on exposure time in 6M KOH solution for LaNi_5-x_Mg_x_ alloys (x = 0, 0.2, and 0.3).

The changes in the polarization resistance value as a function of time, and thus the corrosion rate, were most pronounced for the starting LaNi_5_. After a short one-hour exposure time in solution, the LaNi_5_ electrode showed the highest polarization resistance value, and therefore the lowest corrosion rate (0.033 mA cm^−2^) compared to the magnesium-substituted alloys (Figure 4). Nonetheless, upon prolonged contact with the electrolyte, the corrosion resistance of LaNi_5_ dropped sharply, and after 66 h the corrosion rate increased to 0.91 mA cm^−2^. For a short exposure time of 1 h, the substitution of a part of the nickel with magnesium resulted in a decrease in the polarization resistance, and thus an increase in the corrosion rate, which was especially pronounced for the high-magnesium alloy. However, as can be seen in Figure 4, the corrosion rate for this material did not change significantly over the examined period of time (66 h), and ranged from 0.47 to 0.54 mA cm^−2^. Thus, the longer the contact of the electrolyte with the studied material, the more advantageous the presence of magnesium in the La(Ni, Mg)_5_ alloys became from the point of view of corrosion resistance, especially for the high-magnesium alloy.

**Figure 5:**
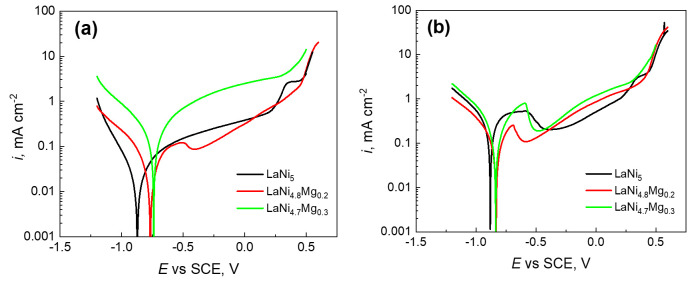
Polarization curves for the three studied alloys. Measurements were performed after 1-hour (**a**) and 66-hour (**b**) exposure of samples in 6M KOH solution.

Figure 5 presents the polarization curves of the LaNi_5_ starting alloy and the LaNi_4.8_Mg_0.2_ and LaNi_4.7_Mg_0.3_ alloys. The measurements were made at time intervals of 1 h and 66 h. When analysing the curves of the potentiokinetic curves after 1 h of exposure, it can be seen that the high-magnesium alloy LaNi_4.7_Mg_0.3_ exhibited the lowest passivation tendency (the values of the passive currents for the LaNi_4.7_Mg_0.3_ alloy were the highest). Nevertheless, after a long exposure time in a corrosive solution (66 h), an improvement in the corrosion resistance of the magnesium-containing alloys was noticed, as evidenced by the width of the passive range, which was greater for alloys containing Mg in relation to the LaNi_5_ starting sample and a decrease of about 2–3 times in the anode currents for the high-magnesium alloy LaNi_4.7_Mg_0.3_, which was attributed to oxidation and the formation of a passive layer on the surface of the alloys, which possibly were enriched in magnesium oxide/hydroxide. As can be seen in Figure 5 for the LaNi_5_ alloy, the slope of the rectilinear section of the cathode curve changed with the increase in the sample exposure time in 6M KOH. The value of the *b*_k_ coefficient increased from 170 to 380 mV/decade, which proved the continuous oxidation of the LaNi_5_ electrode surface. In the case of alloys containing magnesium, the slope of the cathode curve after 1 h of exposure in a corrosive solution was the same as that after 66 h, and amounted to 380 mV/decade. Oxidation of the surface of the electrode materials led to a decrease in their activity and, at the same time, to an increase in the value of the *b*_k_ coefficient.

The research results presented in [13] showed that the corrosion products of MmNi_5_-based alloys in alkaline environments were mainly Mm(OH)_3_. In addition, very active Mg will oxidise to MgO/Mg(OH)_2_ [38]. As known from the literature data, the formation of oxides on the surface of hydrophilic alloys may be a barrier to the penetration of hydrogen into the materials, and can contribute to the deterioration of their hydrogen capacity [13,39,40]. On the other hand, many studies have shown that a corrosion layer consisting of a mixed layer of magnesium oxide and hydroxide had an increased catalytic activity towards the hydrogen evolution reaction [41,42]. In other words, the corrosion layer formed during the corrosion of Mg and its alloys promoted faster H_2_ evolution compared to noncorroded areas of the material, which should have contributed to improvement of the hydrogen capacity of the La(Ni,Mg)_5_ alloys.

**Figure 6:**
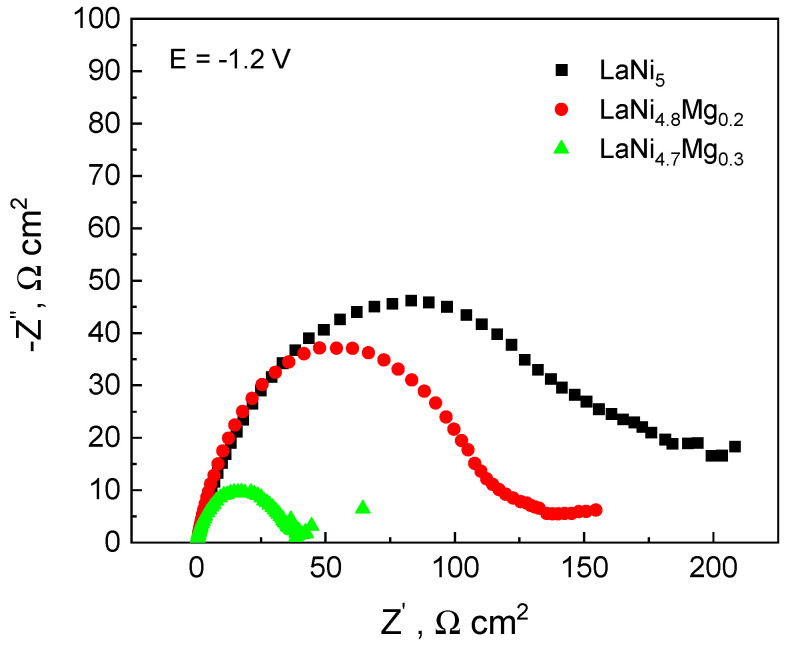
Electrochemical impedance spectra of the LaNi_5-x_Mg_x_ electrodes obtained at a potential of −1.2V. Experimental conditions: 6M KOH, 25 °C, frequency range of 100 kHz to 10 MHz, amplitude of 5 mV.

Figure 6 shows the EIS of the LaNi_5-x_Mg electrodes obtained at the potential of −1.2 V. It can be seen that the diameter of the semicircle in the high-frequency area decreased with the increase in the amount of magnesium in the alloy, which meant a decrease in the charge transfer resistance; i.e., a faster electrochemical reaction. The low resistance favourable to hydrogen absorption/desorption during the charge/discharge processes may have been a consequence of the enrichment of the passive layer (containing La_2_O_3_ and NiO) with MgO oxide, which is known for its low compactness, adhesion to the substrate, and porosity [43]. 

**Figure 7:**
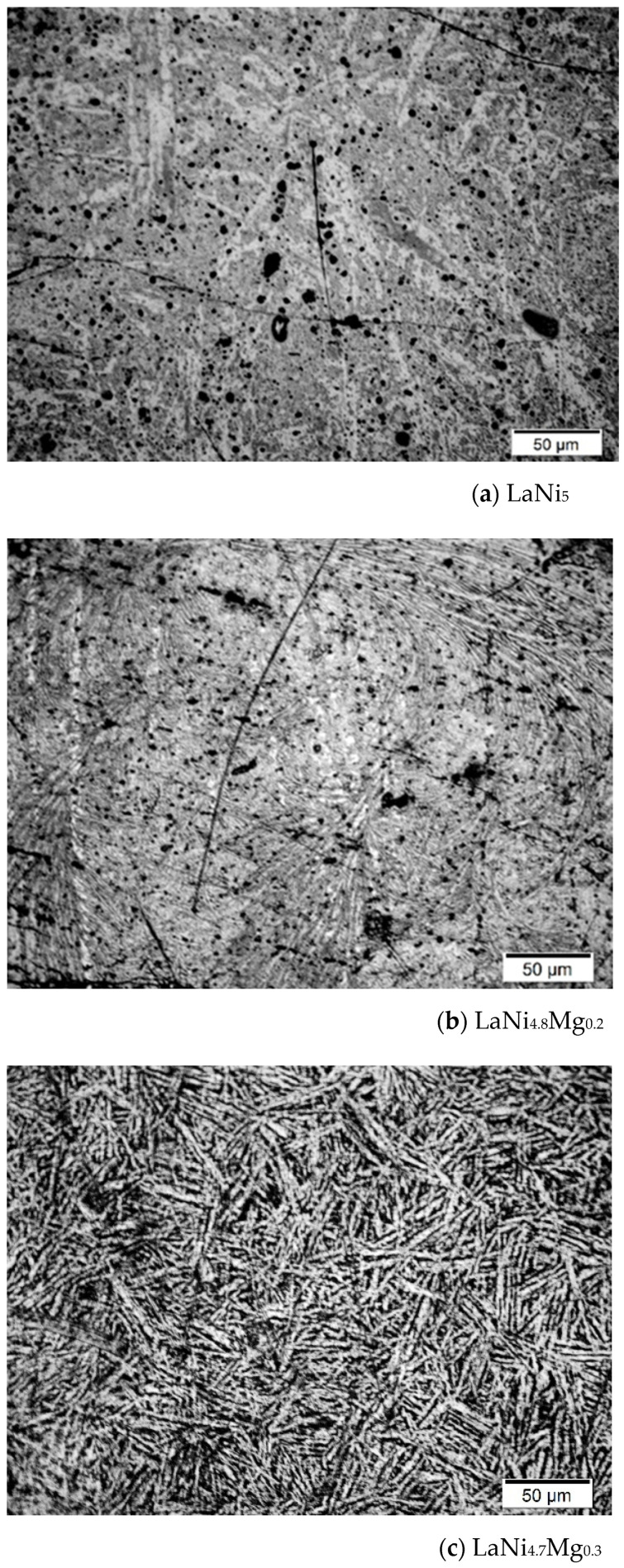
Surface morphology of LaNi_5-x_Mg_x_ alloys after 120 h of exposure in 6M KOH at open circuit potential.

Finally, it was worth paying attention to the qualitative aspect of the surface appearance of the investigated alloys after exposure to a strong alkaline solution (Figure 7). The LaNi_5_ alloy sample was characterised by inhomogeneities (damage and cracks). Passivation in a 6M KOH solution of the low-magnesium alloy (Figure 7b) did not cause large changes in the surface condition; i.e., traces of microcracks were still visible, but there were also areas with a different, more acicular morphology that did not completely cover the observed surface. The surface of a high-magnesium sample after exposure to a corrosive solution was homogeneously (evenly) covered with a layer of acicular morphology. After passivation, the solution showed a greater amount of acicular precipitates than the previous samples (Figure 7c). The improvement of the anticorrosion properties of the LaNi_4.7_Mg_0.3_ alloy, as can be seen in Figure 7, was the result of differences in morphology and the degree of surface coverage by corrosion layers.

## 4. Conclusions

The positive effect of magnesium on the anticorrosive properties of lanthanum and nickel-based alloys was visible after prolonged exposure of the studied electrode materials in an alkaline electrolyte solution. The unfavourable corrosion behaviour of the alloys was influenced by the condition of their surfaces, resulting in an increase in the current density in the passive range. The corrosion rate of the LaNi_5_ alloy increased rapidly after prolonged contact with the electrolyte, in contrast to the Mg-containing alloys, for which the corrosion rate did not change significantly with time; after 66 h, it was about 2–3 times lower than for LaNi_5_. The reason for the significant improvement in the corrosion resistance of the investigated alloys with the addition of magnesium probably was the formation of a fairly tight passive layer enriched with magnesium oxide/hydroxide.

Our conducted research on La(Ni,Mg)_5_ alloys showed promising results for these materials regarding their use in the production of electrodes used in nickel–metal hydride (Ni-MH) cells. The noticeable improvement in the anticorrosion properties of the examined magnesium alloys, which was visible after longer exposure times in a corrosive solution, may contribute to extending the life cycle of Ni-MH cells. Nonetheless, in order to more accurately determine the suitability of these alloys as materials in negative hydride electrodes, additional electrochemical tests should be carried out that allow parameters other than corrosion resistance to be determined, which would be important from the point of view of the application of these alloys.

## Figures and Tables

**Table 1:** Measurement results for chemical composition of LaNi_5-x_Mg_x_ (x = 0, 0.2, and 0.3).

Stop	La (atom %)	Ni (atom %)	Mg (atom %)
LaNi_5_	16.89	83.11	-
	16.78	83.22	-
	16.77	83,23	-
LaNi_4.8_Mg_0.2_	17,13	79.70	3.17
	17.04	79.17	3.79
	17.09	79.62	3.29
LaNi_4.7_Mg_0.3_	18.39	77.42	4.19
	18.54	77.34	4.12
	18.70	76.55	4.75

## Data Availability

The data presented in this study are available upon request from the corresponding author.
